# Long-Term Crop Rotation Revealed the Relationship Between Soil Organic Carbon Physical Fraction and Bacterial Community at Aggregate Scales

**DOI:** 10.3390/microorganisms13030496

**Published:** 2025-02-24

**Authors:** Xianghai Meng, Baicheng Wang, Xingzhe Zhang, Chunguang Liu, Jinghong Ji, Xiaoyu Hao, Bing Yang, Wenhui Wang, Dehai Xu, Shuai Zhang, Xiaomei Wang, Minghui Cao, Yuming Wang

**Affiliations:** 1 Mudanjiang Branch, Heilongjiang Academy of Agricultural Sciences, Mudanjiang 157000, China; mengxianghai538@163.com (X.M.); 13946368993@163.com (B.W.); jxnczxz@163.com (X.Z.); 18045341845@163.com (C.L.); yangb19961112@163.com (B.Y.); wangwenhui1411@126.com (W.W.); 13946350000@163.com (D.X.); 14311@163.com (S.Z.); wxmaiwyp2006@126.com (X.W.); 2 Heilongjiang Academy of Black Soil Conservation & Utilization, Harbin 150086, China; jinghong_98@163.com (J.J.); xiaoyuhao1981@sina.com (X.H.); 3 The Centre for Ion Beam Bioengineering Green Agriculture, Hefei Institutes of Physical Science, Chinese Academy of Sciences, Hefei 230031, China; 4 Science Island Branch, Graduate School, University of Science and Technology of China, Hefei 230026, China

**Keywords:** aggregate, bacterial community, long-term crop rotation, soil organic carbon

## Abstract

Crop rotation enhances soil fertility and health by modulating microbial communities, with soil organic carbon (SOC) dynamics governed by aggregate–microbial interplay. To date, the effects of different crop rotations on SOC fractions and relevant bacterial communities at aggregate scales remain uncertain. Here, a 17-year field experiment was used to reveal the effects of maize monoculture (MM), soybean monoculture (SS), and maize and soybean rotation on the SOC fractions and bacterial communities. Compared with the SS treatment, only the MS treatment significantly increased the particulate organic carbon (POC) content at the aggregate scale. Nevertheless, higher mineral-associated organic carbon (MaOC) contents were observed under the MS and MM treatments than under the SS treatment. The microbial co-occurrence networks for macro- and microaggregates were divided into three main ecological clusters. The specific taxa in Cluster 1 and Cluster 2 are involved in SOC fraction turnover within macro- and microaggregates, respectively. In total, the Vicinamibacteraceae-driven Cluster 1 community dominated the MaOC turnover process within macroaggregates, whereas the Actinobacteria- and Pyrinomonadaceae-driven Cluster 2 communities changed the MaOC turnover process within microaggregates. This study strengthens our understanding of the role of the microbial community in the accumulation of SOC fractions under different crop rotation practices.

## 1. Introduction

Soil organic carbon (SOC) is a fundamental indicator of soil health [1] that reflects not only soil fertility levels [2] but also plays an essential role in achieving sustainable agricultural development and global C neutrality [3]. In general, the accumulation and depletion of SOC are regulated by a combination of aggregates and microorganisms [4]. Soil aggregates are defined as physical structural units in soil that significantly influence SOC turnover [5]. Good aggregate structures offer physical protection and binding sites for SOC [3]. Accordingly, these two SOC fractions are designated POC and MaOC [6]. In contrast, MAOC is subsequently adsorbed by soil minerals and represents the recalcitrant SOC fraction [7]. POC serves as the foundation for the formation of aggregate structures, acting as a cementing agent to facilitate the formation of soil particles and microaggregates into macroaggregates [6]. Consequently, the accumulation and depletion of SOC are also inevitably accompanied by the formation and disintegration of aggregates [8]. The promotion of POC and MAOC accumulations represents a significant strategy for enhancing soil structure and productivity.

On the other hand, soil microbes play a significant role in regulating SOC and aggregate turnover [3,9]. The soil continuum model indicates that the input of organic materials is the premise of SOC formation [10]. The decomposition of exogenous organic materials by soil microorganisms occurs rapidly upon their application to the soil [11]. Some undecomposed and semi-decomposed products, which are the main sources of POC, can be encapsulated by soil aggregates [12]. The further decomposition of these organic materials results in the adsorption of their small biopolymers and C monomer substances by soil minerals, leading to the formation of MaOC [13]. Additionally, some studies have established a relationship between microorganisms and SOC fractions [4,7,14]. The aforementioned factors can be further subdivided into the following categories: microbial species, clusters, and community complexity [4,15,16]. However, previous studies have revealed significant differences in microbial community compositions because environmental factors such as water, nutrients, specific surface areas, and oxygen are different in macro- and microaggregates [4,7]. For example, a previous study demonstrated that fungi were more effectively enriched in macroaggregates, whereas bacteria were predominantly present in the silt–clay fraction [17,18]. Additionally, Bach et al. reported that Actinomycetes were more abundant in microaggregates than in macroaggregates [19]. Consequently, the microbial C sequestration mechanisms were different across the aggregates. To date, few studies have investigated the relationships between microbes and SOC at the aggregate scale.

Crop rotation is an important agronomic practice that is often used to avoid obstacles related to succession cropping [20], improve soil fertility [21], and promote sustainable agriculture [22], the key to which is the regulation of soil microbial communities through changes in root exudate components and straw input levels [23,24]. Endogenous flavonoids facilitate legume–rhizobium symbiosis, and the greater nitrogen fixation efficiency of rhizobia can increase the soil nitrogen content and promote nitrogen uptake by crops [25]. Zhao et al. reported that [26], via a global meta-analysis, legumes enhanced the main crop yields by 20%. Additionally, the cotton verticillium wilt index is influenced by phenolic acids in the root exudates [27]. From this perspective, the construction of a stable and healthy soil microbial community represents a fundamental objective of crop rotation measures.

The Northeast China Plain is a significant grain-producing region in China [28]. However, SOC levels have declined due partly to the practice of frequent plowing and anthropogenic activities that have not been sustainable [29]. In light of the aforementioned evidence, this study conducted a 17-year field trial with the objective of investigating (i) the differences in soil aggregates and organic carbon fractions under different crop rotations and (ii) the relationships among SOC physical fractions and microorganisms at the aggregate scale. We hypothesize that (1) long-term crop rotation promotes SOC fraction accumulation by facilitating the formation of macroaggregates and that (2) crop rotation facilitates the accumulation of SOC fractions by regulating microbial traits at the aggregate scale.

## 2. Materials and Methods

### 2.1. Site Description and Sampling

Initiated in 2006, the experimental site is situated in Wenchun (44°59′61″ N, 129°59′18″ E), Mudanjiang City, within the fertile Northeast China Plains renowned for cereal production. This agroecosystem experiences a temperate continental monsoon climate characterized by distinct seasonal variations, with long-term records indicating mean annual values of 5.0 °C for temperature and 579.7 mm for precipitation. The predominant soil type was identified as meadow soil according to the US Soil Taxonomy system. The cropping system consisted of a continuous maize (*Zea mays* L.) monoculture (MM), soybean (*Glycine max* L.) monoculture (SS), and maize and soybean and rotation (MS).

The fertilization strategy during the maize season included 84.00 kg N/ha, 40.17 kg P/ha, and 62.23 kg K/ha, and the fertilization strategy during the soybean season included 50.00 kg N/ha, 43.66 kg P/ha, and 22.41 kg K/ha. Generally, N is applied in the form of urea (N 46%), and P and K are applied in the form of diammonium phosphate (P_2_O_5_ 46%) and potassium chloride (K_2_O 60%).

Soil samples were collected from the 0–20 cm layer in October 2023 post-maize harvest. The experiment utilized a randomized complete block design with three treatments (n = 3). Each plot (7.8 m × 20 m) was sampled using nine 5 cm diameter soil cores composited per replicate. Composite soil samples were formed by mixing nine randomly collected subsamples (5 cm diameter) per plot. The experimental design (3 treatments × 3 replicates) yielded 9 samples total. Field-moist samples were stored in rigid containers at 4 °C with 40% moisture for 72 h to preserve aggregate integrity. Prior to analysis, samples were sieved (8 mm) with manual removal of roots and mineral debris. Subsamples were split into two aliquots: air-dried for basic characterization and fresh for aggregate fractionation.

### 2.2. Basic Chemical Properties of the Soil

Soil pH was determined electrometrically at a 1:2.5 (*w*/*w*) soil–water ratio following standard protocols. SOC content was quantified via dichromate oxidation titration, while total N was analyzed using the Kjeldahl method. P fractions were measured by molybdenum blue colorimetry, and K levels (total/available) were assessed via flame photometry [30]. Pre- and post-treatment soil chemical characteristics for the various crop rotation regimes are shown in Table S1.

### 2.3. Soil Aggregate Isolation and SOC Fraction Determination

Soil aggregates were fractionated using Six et al.’s physical separation protocol [31], yielding macroaggregates (0.25–2 mm), microaggregates (0.053–0.25 mm), and silt–clay fractions. Particulate organic carbon (POC) and mineral-associated OC (MaOC) were isolated via Yu et al.’s chemical dispersion method [32]. All aggregate C levels were measured using K_2_Cr_2_O_7_ digestion [30]. Detailed testing methods are described in Text S1.

The mean weight diameter (MWD) was used to describe the soil physical structure and was calculated via the following formula:

MWD = ∑Xi × Wi
(1)

where Xi represents the average diameter of each aggregate size and Wi represents the proportion of each aggregate weight relative to the total sample weight after wet sieving. The upper limit for the macroaggregate diameter was 2 mm.

### 2.4. DNA Extraction and 16S rRNA Sequencing at Aggregate

Subsamples were freeze-dried post-wet sieving for DNA extraction. Total DNA was isolated from 0.5 g aliquots using a commercial soil-specialized kit (FastDNA^TM^ Spin Kit, MP Biomedicals, Santa Ana, CA, USA) following manufacturer protocols, with triplicate biological replicates per treatment. Purified DNA was cryopreserved (−80 °C) prior to downstream analysis.

Bacterial 16S rRNA genes (V4–V5) were amplified using primers 515F (5′-GTGCCAGCMGCCGCGGTAA-3′) and 907R (5′-CCGTCAATTCMTTTRAGTTT-3′), followed by paired-end sequencing on an Illumina MiSeq platform [33]. Specific protocols for PCR amplification, library preparation, and quality control are detailed in Text S2.

### 2.5. Statistical Analysis

Prior to analysis, data normality and variance homogeneity were assessed using Kolmogorov–Smirnov and Levene’s tests, respectively. Non-normal data underwent log or square-root transformations. Treatment effects on soil properties and microbial indicators were analyzed through one-way ANOVA (SPSS 21.0, SPSS Inc., Chicago, IL, USA) with Duncan’s multiple-range test (α = 0.05).

Shannon indices were computed using the “vegan (2.6.8)” package in R (4.2.2). Bacterial community structure shifts were assessed through principal co-ordinate analysis (PCoA), with treatment differences confirmed by Adonis test. Microbial interaction networks were built with “igraph (2.1.1)” and “WGCNA (1.73)” packages, retaining taxa > 0.05% relative abundance and applying Pearson’s correlation thresholds (|*r*| > 0.8, *p* < 0.05). Networks were visualized via Gephi 0.10, with topological metrics calculated [34]. MENA2 categorized nodes as peripherals (*Zi* ≤ 2.5, *Pi* ≤ 0.62), connectors (*Zi* ≤ 2.5, *Pi* > 0.62), cluster hubs (*Zi* > 2.5, *Pi* ≤ 0.62), and network hubs (*Zi* > 2.5, *Pi* > 0.62) [34,35]. Aggregate-scale SOC fractions and keystone taxa relationships were visualized in heatmaps generated using Origin 2021.

## 3. Results

### 3.1. Soil Aggregate Distributions and SOC Fraction Contents Under Different Crop Rotations

Long-term crop rotation practices significantly influence soil aggregate distributions (Table 1). Compared with crop monocultures (MM and MS treatments), the MS treatment significantly increased the percentage of macroaggregate material (*p* < 0.05), whereas the percentage of microaggregate material under the SS treatment was greater than under the MM and MS treatments (*p* < 0.05). The highest percentages of the silt and clay fractions were observed under the MM treatment, which were significantly greater than those under the SS and MS treatments (*p* < 0.05). The mean weight diameter (MWD) was used to evaluate the stability of the aggregate structure. Table 1 indicates that the MWD was greater under the MS treatment than under the other treatments (*p* < 0.05).

**Figure 1:**
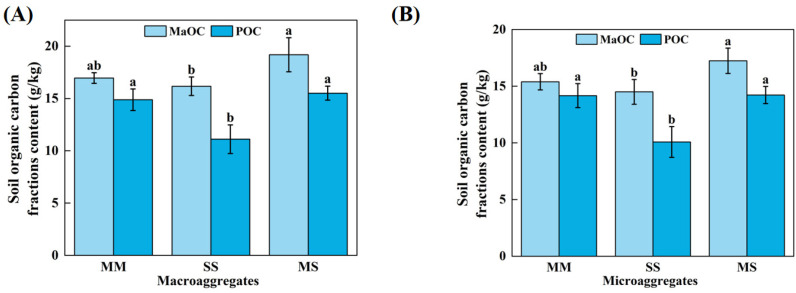
Soil organic carbon fractions content within macroaggregate (**A**) and microaggregate (**B**) under different treatments. POC, particulate organic carbon; MaOC, mineral-associated organic carbon; MM, maize monoculture; SS, soybean monoculture; MS, maize–soybean rotation. Different letters indicate a significant difference (*p* < 0.05).

Moreover, contrasting cropping patterns significantly altered the contents of SOC compared to pre-experiment conditions (Table S1). To elucidate the mechanisms underlying these changes, we conducted a comprehensive analysis of SOC fractions at the aggregate scale (Figure 1 and Figure S1). Overall, the SOC, POC, and MaOC contents tended to decrease in the order MS > MM > SS. In the bulk soil and aggregates, the SOC contents were significantly greater in the MS treatment than in the SS treatment (*p* < 0.05, Figure S1). For both macro- and microaggregates, the POC content under the MS treatment was significantly greater than that under the SS treatment (*p* < 0.05, Figure 1), and the MaOC contents under the MS and MM treatments were significantly greater than that under the SS treatment (*p* < 0.05, Figure 1). In addition, the POC and MaOC contents in macroaggregates were greater than those in microaggregates. Therefore, compared with crop monocultures, crop rotation improved the aggregate structure stability and SOC accumulations.

### 3.2. Soil Microbial Diversities and Compositions at Various Scales

Long-term crop rotation practices also altered bacterial diversity (Table S2 and Figure S2). There was no significant difference in bacterial alpha diversity between the treatments (Table S2). However, the bacterial communities changed under the different treatments (Figure S2). The first two axes explained 42.85% and 51.00% of the variance in the bacterial community in the macro- and microaggregates, respectively. Although the soil microbial communities changed under the different cropping systems, Acidobacteria, Proteobacteria, Acidobacteria, and Chloroflexi were the main phyla of bacteria, accounting for more than 85% of the total bacterial abundance (Figure S3).

### 3.3. Linkages Between SOC Fractions and Microbial Traits at Aggregate Scales

**Figure 2:**
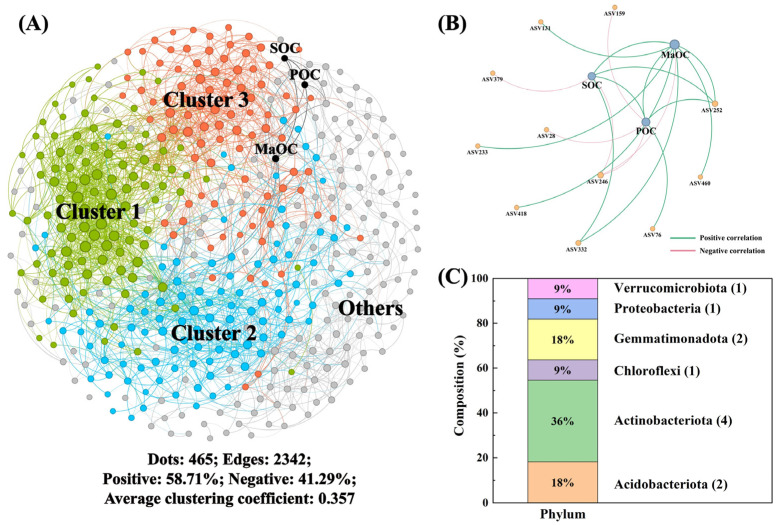
The relationships of microbial taxa with SOC fractions within macroaggregate. Soil microbial co-occurrence network (**A**), the associations of microbial taxa with SOC (**B**), and the phylum of selected bacterial taxa (**C**). Different colors represent different clusters.

**Figure 3:**
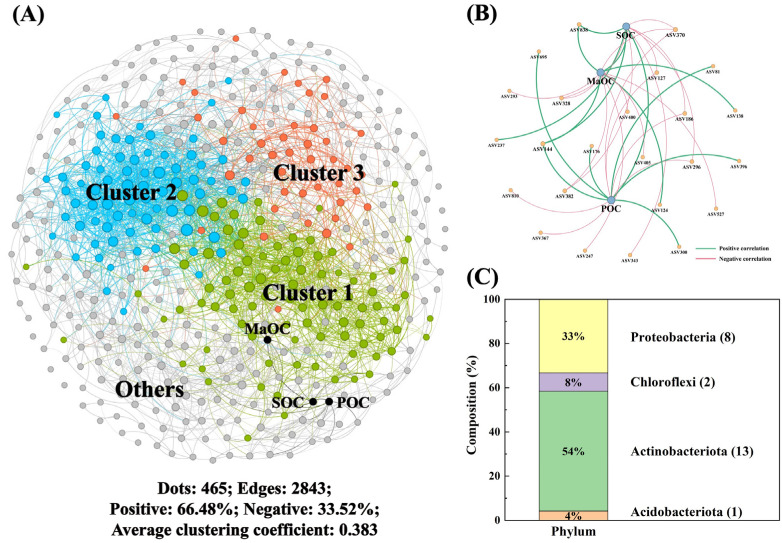
The relationships of microbial taxa with SOC fractions within microaggregate. Soil microbial co-occurrence network (**A**), the associations of microbial taxa with SOC (**B**), and the phylum of selected bacterial taxa (**C**). Different colors represent different clusters.

Two co-occurrence networks were constructed at the macroaggregate (Figure 2) and microaggregate (Figure 3) scales to evaluate the associations between specific bacterial taxa and SOC fractions. The macroaggregate co-occurrence network contained 465 nodes and 2342 edges (58.71% were positively correlated), which were divided into three main clusters (Figure 2A). Furthermore, we selected the specific taxa that were closely associated with the SOC fractions (Figure 2B), and the main bacterial taxa were Actinobacteriota (36%), Acidobacteriota (18%), and Gemmatiomonadota (18%) (Figure 2C). After further classification, the potential SOC turnover bacterial species belonged to the Cluster 1 community (Table S3). For some specific taxa, the relative abundances of Streptomyces and Vicinamibacteraceae within the Cluster 1 community were positively correlated with the POC and MaOC contents, respectively (Table S3).

Similarly, for the microaggregates, the co-occurrence network contained 465 nodes and 2843 edges (66.48% were positively correlated), which were divided into three main clusters (Figure 3A). Furthermore, we selected the specific taxa that were closely associated with the SOC fractions (Figure 3B), and the main bacterial taxa were Actinobacteria (54%) and Proteobacteria (33%) (Figure 3C). After further classification, the potential SOC turnover bacterial species belonged to Cluster 2 communities (Table S4). For some specific taxa, such as *Saccharothrix* and Enterobacteriaceae, their relative abundances were positively correlated with the POC content, whereas the relative abundances of *Saccharothrix* and Pseudomonas were positively correlated with the MaOC content (Table S3). Considering the number of edges and the proportion of positive correlations, the stability of microbial communities in microaggregates is greater.

**Figure 4:**
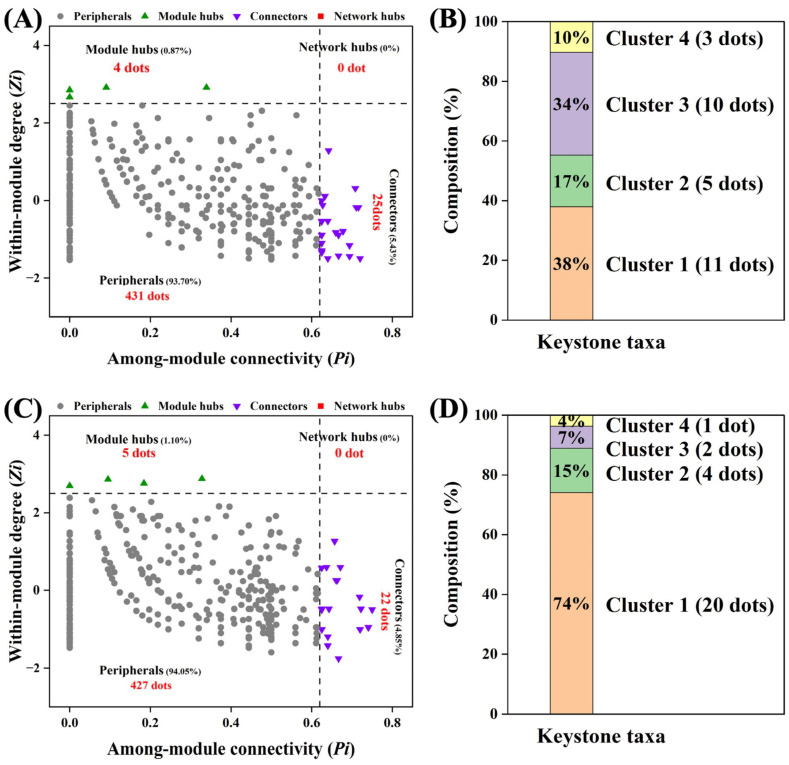
ZP plot showing distribution of ASVs based on their module-based topological roles. The topological role of each OTU was determined according to the scatter plot of within-module connectivity (*Zi*) and among-module connectivity (*Pi*). Topological roles within macroaggregates (**A**) and microaggregates (**C**). The keystone taxa compositions within macroaggregates (**B**) and microaggregates (**D**).

**Figure 5:**
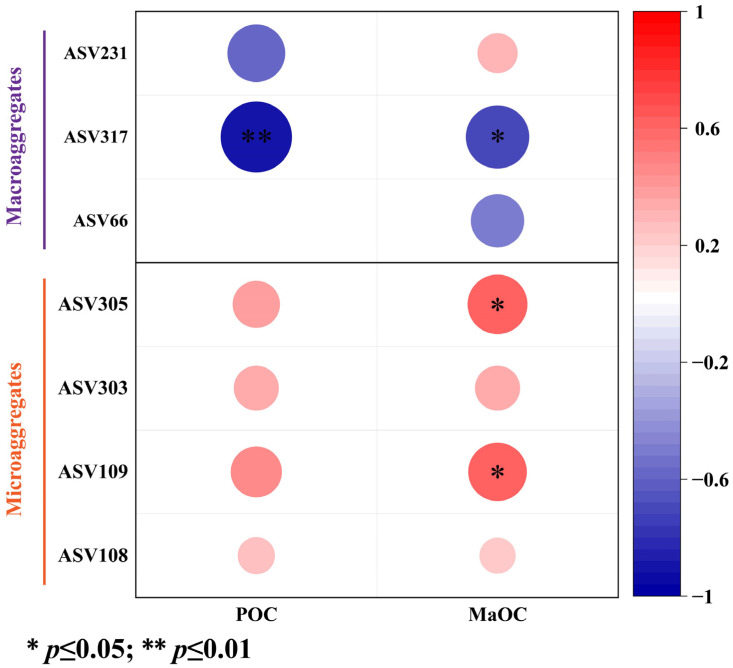
Heatmap revealing the correlation coefficients between keystone taxa with SOC fractions. POC, particulate organic carbon; MaOC, mineral-associated organic carbon.

ZP plots were constructed to identify the topological roles of each node in the network. As shown in Figure 4A, in macroaggregates, a total of 29 bacterial taxa were identified as keystone species, of which 25 belong to connectors and 4 belong to cluster hubs. The selected species were identified mainly in Cluster 1 and 3 communities (Figure 4B). In macroaggregates, a total of 27 bacterial taxa were identified as keystone species, of which 22 belong to connectors and 5 belong to cluster hubs. The selected species were identified mainly in Cluster 1 and 2 communities (Figure 4D). The correlations between keystone taxa and SOC fractions within aggregates are shown in Figure 5 and Table S4. The relative abundance of Vicinamibacteraceae (within the Cluster 1 community) was negatively correlated with the SOC fraction content in macroaggregates, whereas the relative abundances of Actinobacterium and Pyrinomonadaceae (within the Cluster 2 community) were positively correlated with the MaOC content in microaggregates.

## 4. Discussion

### 4.1. Long-Term Crop Rotation Promotes POC Accumulation by Facilitating Aggregate Stability

The stability of soil aggregates can influence the retention of soil water, aeration, nutrient accumulation, and SOC turnover [1,36,37]. Consequently, the aggregate structure stability is highly important for soil fertility and serves as a key indicator for assessing soil health [37]. As anticipated (Hypothesis 1), long-term crop rotation significantly increased the proportion of macroaggregates as well as the aggregate stability (Table 1). The aggregate stability is typically influenced by a number of factors, including tillage practices, climate, irrigation, straw return, and crop type [3,23,38,39,40,41,42]. In a single controlled field environment, macroaggregate formation is influenced primarily by the greater biomass input to the field, which provides more organic binders for soil macroaggregate formation, thereby promoting its stability [43]. Furthermore, crop roots are also a significant contributing factor to the process of aggregate formation [8]. Compared with soybean roots, maize roots are more developed and can secrete more exudates [44]. Moreover, the entanglement effect of the roots can also promote aggregate stability [45]. Consequently, the proportion of macroaggregates and aggregate stability are clearly enhanced under conditions of maize–soybean rotation and maize monoculture.

Furthermore, our study indicated that the aggregate stability remained 12.81% greater in the maize–soybean rotation system than in the maize monoculture system (Table 1). This finding also indicates that a diverse cropping pattern has a beneficial influence on aggregate stability. The results of the meta-analyses conducted in previous studies indicated that, compared with crop monocultures, crop rotation generally increased aggregate stability by 15–35% [46,47]. This finding is consistent with the results of the present study (Table 1). The diversity of straw substrates may serve as a complementary C source for the soil [23,48]. For example, the elevated N content of soybean straw resulted in a reduction in the soil C/N ratio (Table S1), thereby facilitating the rapid formation of organic matter [49]. In addition, diverse cropping patterns can alter the aggregate formation process by regulating soil microbial communities [50]. Maize–soybean rotation systems provide abundant and varied C and N sources for microorganisms (Table S1), promoting microbial diversity and abundance, facilitating the rapid degradation of exogenous materials and increasing aggregate stability [51].

Numerous studies have investigated the relationship between aggregates and SOC [3,4,52]. In general, the formation of macro- and microaggregates through soil cementation involves the aggregation of silt and clay particles [53]. Consequently, as the size of the aggregates decreased, the SOC contents also decreased, which is consistent with the results of this study (Figure 1). The formation of macroaggregates provides physical protection for POC, thereby reducing its further decomposition and utilization by crop roots and microorganisms and preventing the loss of POC [8]. Therefore, the diverse straw substrates used in the maize–soybean rotation promoted aggregate stability, thus providing favorable conditions for POC accumulation.

### 4.2. Long-Term Crop Rotation Promotes Accumulation of SOC Fractions by Regulating the Microbial Community at Aggregate Scales

Soil microbes play a pivotal role in the decomposition of exogenous organic matter and the turnover of SOC [3,54]. Soil continuum modeling indicates that small polymers and C monomers that can be adsorbed by soil minerals must undergo microbial decomposition [53]. However, owing to the differing macro- and microaggregate environments, the microbial mechanisms of action for SOC accumulation are necessarily distinct [55]. Accordingly, in the present study, the microbial co-occurrence network was initially employed to identify the microorganisms directly involved in the SOC fraction [3]. First, a comparison of the microbial communities at the aggregate scale revealed that the microbial community structure was more stable in microaggregates (Figure 2A and Figure 3A). This finding is consistent with the results of previous studies, which have demonstrated that macroaggregates are enriched with more fungi, whereas bacteria are more likely to dominate as the aggregate size decreases [19]. Furthermore, bacteria are more likely to be involved in SOC turnover in microaggregates (Figure 2B and Figure 3B). In macroaggregates, the bacterial species identified in the Cluster 1 and 3 communities demonstrated greater correlations with the SOC fractions (Table S3). Among these bacterial species, *Streptomyces*, Vicinamibacteraceae, Pedosphaeraceae, and Gemmatimonadaceae presented significant positive correlations with MaOC. Schlatter et al. reported that *Streptomyces* demonstrated a broad range of efficient exploitation of C sources [56]. Yang et al. reported that Vicinamibacteraceae functions in chemoheterotrophy [57]. The genomes of Gemmatimonadaceae have been demonstrated to be enriched with genes responsible for benzoate and styrene degradation [58]. The decomposed components are also the main source of MaOC. The Pedosphaeraceae are enriched mainly in the rhizosphere and are able to promote plant metabolism [59,60]. Therefore, the maize–soybean rotation provided a favorable living environment for these bacterial species, which can promote the secretion of root exudates by plants and thorough straw degradation.

In the microaggregates, the bacterial species identified in the Cluster 2 community demonstrated greater correlations with the SOC fractions (Table S4). *Saccharothrix* is a typical phosphorus-solubilizing bacterial strain with a high level of stress resistance in the environment [61]. Owing to the limitations of spatial and nutrient availability in microaggregates, *Saccharothrix* has become the dominant strain and can take up nutrients from straw [62]. During this process, the decomposition products of straw gradually became the sources of POC and MaOC [3]. Enterobacteriaceae are considered important species with the ability to decompose organic materials, which are enriched in loam clay and sandy material [63]. Additionally, *Pseudomonas* has been frequently investigated as a potential antagonist for soil-borne diseases and has also been demonstrated to facilitate SOC turnover by utilizing humic acid [64]. The microscopic residues of plants and animals represent the primary sources of humic acid, which also indicates the capacity of *Pseudomonas* to decompose exogenous materials and promote MaOC accumulation [65].

To verify the correctness of the above results, keystone species were further screened (Figure 4 and Figure 5). Microbial keystone species also have great explanatory power in terms of network structure and function [66,67,68]. In macroaggregates, the keystone species belong mainly to Cluster 1. Surprisingly, Vicinamibacteraceae was significantly correlated with the SOC fraction, which is consistent with our results above [69]. Among the microaggregates within Cluster 2, *Actinobacterium* are traditional and typical straw-degrading bacteria that have been widely studied and are enriched in microaggregates [70,71]. Lee et al. reported that Pyrinomonadaceae has strong C use efficiency and can promote its accumulation [72]. In summary, there are differences in the microbial mechanisms of SOC sequestration between different aggregate particle sizes. Maize–soybean rotation can promote SOC accumulation by regulating microbial communities.

## 5. Conclusions

The maize–soybean rotation enhances SOC dynamics through synergistic aggregate–microbial interactions. Elevated crop-derived carbon inputs stimulate microbial proliferation and enzymatic activity, accelerating exogenous organic matter decomposition while enhancing macroaggregate formation via organic cementation. These macroaggregates physically protect POC, while microbial processing converts residual substrates into monomeric C forms that mineral-adsorb to form MaOC.

The implementation of distinct cropping patterns resulted in notable alterations in the soil aggregate distribution and content of SOC fractions. The proportions of soil macroaggregates and the MWD were greater under maize–soybean rotation, which promoted the accumulation of POC fractions at the aggregate scale. Furthermore, the relationships between soil microbes and SOC are variable at the aggregate scale. In macroaggregates, the specific taxa within Cluster 1 were involved in SOC fraction turnover. The richness of Vicinamibacteraceae and *Streptomyces* in Cluster 1 was significantly positively correlated with MaOC, and the richness of *Streptomyces* was significantly positively correlated with POC. In microaggregates, bacterial Cluster 2 communities mainly participate in SOC fraction turnover. The richness of *Saccharothrix* and Enterobacteriaceae promoted POC accumulation, whereas *Saccharothrix*, *Pseudomonas*, and *Actinophytocola* promoted MaOC accumulation. Therefore, long-term maize–soybean rotation is a reliable strategy for maintaining soil fertility and productivity on the Northeast China Plain.

## Figures and Tables

**Table 1:** The aggregate distribution and stability under different treatments.

Treatments	Macroaggregate (%)	Microaggregate (%)	Silt and Clay (%)	MWD (mm)
MM	62.07 ± 2.17 b	23.51 ± 2.53 b	14.42 ± 0.60 a	73.78 ± 2.07 b
SS	58.59 ± 2.29 b	36.99 ± 3.05 a	4.43 ± 0.86 c	71.63 ± 2.14 b
MS	70.96 ± 10.57 a	21.01 ± 5.76 b	8.02 ± 4.90 b	83.23 ± 10.90 a

The results show means ± standard deviations (n = 3). Different lowercase letters after values indicate significant differences between each treatment, *p* < 0.05. MM, maize monoculture; SS, soybean monoculture; MS, maize–soybean rotation.

## Data Availability

The original contributions presented in the study are included in the article/Supplementary Material, further inquiries can be directed to the corresponding authors.

## Supplementary Materials

The following supporting information can be downloaded at: https://www.mdpi.com/article/10.3390/microorganisms13030496/s1, Text S1: Soil aggregate isolation and SOC fraction determination; Text S2: High-throughput sequencing; Table S1: The soil basic property under different treatments; Table S2: The soil microbial shannon index under different treatments; Table S3: The information of selected bacterial taxa within macroaggregate; Table S4: The information of selected bacterial taxa within microaggregate; Table S5: The information of microbial keystone taxa; Figure S1: Soil organic carbon content in bulk soil (A), macroaggregate (B) and microaggregate (C). SOC, soil organic carbon. MM, maize monoculture; SS, soybean monoculture; MS, maize-soybean rotation; Figure S2: PCoA showing the bacteria within macroaggregate (A) and microaggregate (B) under different treatments. MM, maize monoculture; SS, soybean monoculture; MS, maize-soybean rotation; Figure S3: Soil microbial community compositions within macroaggregate (A) and microaggregate (B) under different treatments. MM, maize monoculture; SS, soybean monoculture; MS, maize-soybean rotation.

## References

[1] Hatano R., Mukumbuta I., Shimizu M. (2024). Soil Health Intensification through Strengthening Soil Structure Improves Soil Carbon Sequestration. Agriculture.

[2] Placek A., Grobelak A., Wloka D., Kowalska A., Singh B.R., Almas A., Kacprzak M. (2018). Methods for Calculating Carbon Sequestration in Degraded Soil of Zinc Smelter and Post-Mining Areas. Desalin. Water Treat..

[3] Duan Y., Chen L., Zhang J., Li D., Han X., Zhu B., Li Y., Zhao B., Huang P. (2021). Long-Term Fertilisation Reveals Close Associations between Soil Organic Carbon Composition and Microbial Traits at Aggregate Scales. Agric. Ecosyst. Environ..

[4] Zhang M., Song X., Wu X., Zheng F., Li S., Zhuang Y., Man X., Degré A. (2024). Microbial Regulation of Aggregate Stability and Carbon Sequestration under Long-Term Conservation Tillage and Nitrogen Application. Sustain. Prod. Consum..

[5] Yudina A., Kuzyakov Y. (2023). Dual Nature of Soil Structure: The Unity of Aggregates and Pores. Geoderma.

[6] Even R.J., Cotrufo M.F. (2024). The Ability of Soils to Aggregate, More than the State of Aggregation, Promotes Protected Soil Organic Matter Formation. Geoderma.

[7] Li Z., Duan X., Guo X., Gao W., Li Y., Zhou P., Zhu Q., O’Donnell A.G., Dai K., Wu J. (2024). Microbial Metabolic Capacity Regulates the Accrual of Mineral-Associated Organic Carbon in Subtropical Paddy Soils. Soil Biol. Biochem..

[8] Xiao L., Zhang W., Hu P., Xiao D., Yang R., Ye Y., Wang K. (2021). The Formation of Large Macroaggregates Induces Soil Organic Carbon Sequestration in Short-Term Cropland Restoration in a Typical Karst Area. Sci. Total Environ..

[9] Liang A., Zhang Y., Zhang X., Yang X., McLaughlin N., Chen X., Guo Y., Jia S., Zhang S., Wang L. (2019). Investigations of Relationships among Aggregate Pore Structure, Microbial Biomass, and Soil Organic Carbon in a Mollisol Using Combined Non-Destructive Measurements and Phospholipid Fatty Acid Analysis. Soil Tillage Res..

[10] Lehmann J., Kleber M. (2015). The Contentious Nature of Soil Organic Matter. Nature.

[11] Liu X., Chen Q., Zhang H., Zhang J., Chen Y., Yao F., Chen Y. (2023). Effects of Exogenous Organic Matter Addition on Agricultural Soil Microbial Communities and Relevant Enzyme Activities in Southern China. Sci. Rep..

[12] Si Q., Chen K., Wei B., Zhang Y., Sun X., Liang J. (2024). Dissolved Carbon Flow to Particulate Organic Carbon Enhances Soil Carbon Sequestration. SOIL.

[13] Kleber M., Bourg I.C., Coward E.K., Hansel C.M., Myneni S.C.B., Nunan N. (2021). Dynamic Interactions at the Mineral–Organic Matter Interface. Nat. Rev. Earth Environ..

[14] Wu B., Zhang M., Zhai Z., Dai H., Yang M., Zhang Y., Liang T. (2024). Soil Organic Carbon, Carbon Fractions, and Microbial Community under Various Organic Amendments. Sci. Rep..

[15] Ji L., Tan W., Chen X. (2019). Arbuscular Mycorrhizal Mycelial Networks and Glomalin-Related Soil Protein Increase Soil Aggregation in Calcaric Regosol under Well-Watered and Drought Stress Conditions. Soil Tillage Res..

[16] Hati K.M., Jha P., Dalal R.C., Jayaraman S., Dang Y.P., Kopittke P.M., Kirchhof G., Menzies N.W. (2021). 50 Years of Continuous No-Tillage, Stubble Retention and Nitrogen Fertilization Enhanced Macro-Aggregate Formation and Stabilisation in a Vertisol. Soil Tillage Res..

[17] Rillig M.C., Aguilar-Trigueros C.A., Bergmann J., Verbruggen E., Veresoglou S.D., Lehmann A. (2015). Plant Root and Mycorrhizal Fungal Traits for Understanding Soil Aggregation. New Phytol..

[18] Tiemann L.K., Grandy A.S., Atkinson E.E., Marin-Spiotta E., McDaniel M.D. (2015). Crop Rotational Diversity Enhances Belowground Communities and Functions in an Agroecosystem. Ecol. Lett..

[19] Bach E.M., Williams R.J., Hargreaves S.K., Yang F., Hofmockel K.S. (2018). Greatest Soil Microbial Diversity Found in Micro-Habitats. Soil Biol. Biochem..

[20] Liu C., Plaza-Bonilla D., Coulter J.A., Kutcher H.R., Beckie H.J., Wang L., Floc’h J.-B., Hamel C., Siddique K.H.M., Li L., Sparks D.L. (2022). Chapter Six—Diversifying Crop Rotations Enhances Agroecosystem Services and Resilience. Advances in Agronomy.

[21] Zhang L., Yuan J., Zhang M., Zhang Y., Wang L., Li J. (2022). Long Term Effects of Crop Rotation and Fertilization on Crop Yield Stability in Southeast China. Sci. Rep..

[22] Shah K.K., Modi B., Pandey H.P., Subedi A., Aryal G., Pandey M., Shrestha J. (2021). Diversified Crop Rotation: An Approach for Sustainable Agriculture Production. Adv. Agric..

[23] Duan Y., Cao M., Zhong W., Wang Y., Ni Z., Zhang M., Li J., Li Y., Meng X., Wu L. (2023). Straw Return with Diverse Nitrogen Fertilizer Application Rates Modulate Ecosystem Services and Microbial Traits in a Meadow Soil. EGUsphere.

[24] Liu C., Wang J., Wang Y., Li L., Feng Z., Xian Y., Jiang Y., Yu J., Tong T., Li X. (2024). Crop Rotation and Fertilization Shape the Microbiomes of Maize Rhizosphere Soil with Distinct Mechanisms. Plant Soil.

[25] Qiu X., Wang W., Yang J., Li D., Jiao J., Wang E., Yuan H. (2024). Fulvic Acid Promotes Legume-Rhizobium Symbiosis by Stimulating Endogenous Flavonoids Synthesis and Secretion. J. Agric. Food Chem..

[26] Zhao J., Chen J., Beillouin D., Lambers H., Yang Y., Smith P., Zeng Z., Olesen J.E., Zang H. (2022). Global Systematic Review with Meta-Analysis Reveals Yield Advantage of Legume-Based Rotations and Its Drivers. Nat. Commun..

[27] Zhang G., Raza W., Wang X., Ran W., Shen Q. (2012). Systemic Modification of Cotton Root Exudates Induced by Arbuscular Mycorrhizal Fungi and *Bacillus vallismortis* HJ-5 and Their Effects on Verticillium Wilt Disease. Appl. Soil Ecol..

[28] Ma H., Peng M., Yang Z., Yang K., Zhao C., Li K., Guo F., Yang Z., Cheng H. (2024). Spatial Distribution and Driving Factors of Soil Organic Carbon in the Northeast China Plain: Insights from Latest Monitoring Data. Sci. Total Environ..

[29] Zhao Y., Wang M., Hu S., Zhang X., Ouyang Z., Zhang G., Huang B., Zhao S., Wu J., Xie D. (2018). Economics- and Policy-Driven Organic Carbon Input Enhancement Dominates Soil Organic Carbon Accumulation in Chinese Croplands. Proc. Natl. Acad. Sci. USA.

[30] Lu R.K. (2000). The Analysis Method of Soil Agricultural Chemistry. Chin. Agric. Sci. Technol. Press.

[31] Six J., Elliott E.T., Paustian K. (2000). Soil Macroaggregate Turnover and Microaggregate Formation: A Mechanism for C Sequestration under No-Tillage Agriculture. Soil Biol. Biochem..

[32] Yu H., Ding W., Luo J., Geng R., Cai Z. (2012). Long-Term Application of Organic Manure and Mineral Fertilizers on Aggregation and Aggregate-Associated Carbon in a Sandy Loam Soil. Soil Tillage Res..

[33] Caporaso J.G., Lauber C.L., Walters W.A., Berg-Lyons D., Huntley J., Fierer N., Owens S.M., Betley J., Fraser L., Bauer M. (2012). Ultra-High-Throughput Microbial Community Analysis on the Illumina HiSeq and MiSeq Platforms. ISME J..

[34] Ma J., Gonzalez-Ollauri A., Zhang Q., Xiao D., Chen F. (2021). Ecological Network Analysis to Assess the Restoration Success of Disturbed Mine Soil in Zoucheng, China. Land Degrad. Dev..

[35] Deng Y., Jiang Y.-H., Yang Y., He Z., Luo F., Zhou J. (2012). Molecular Ecological Network Analyses. BMC Bioinform..

[36] Nweke I.A., Nnabude P.C. (2015). Aggregate Stability of Four Soils as Evaluated by Different Indices. J. Exp. Biol. Agric. Sci..

[37] Rieke E.L., Bagnall D.K., Morgan C.L.S., Flynn K.D., Howe J.A., Greub K.L.H., Mac Bean G., Cappellazzi S.B., Cope M., Liptzin D. (2022). Evaluation of Aggregate Stability Methods for Soil Health. Geoderma.

[38] Haynes R., Swift R., Stephen R. (1991). Influence of Mixed Cropping Rotations (Pasture Arable) on Organic-Matter Content, Water Stable Aggregation and Clod Porosity in a Group of Soils. Soil Tillage Res..

[39] Cerdà A. (2000). Aggregate Stability against Water Forces under Different Climates on Agriculture Land and Scrubland in Southern Bolivia. Soil Tillage Res..

[40] Lehrsch G.A., Robbins C.W., Brown M.J. (2008). Whey Utilization in Furrow Irrigation: Effects on Aggregate Stability and Erosion. Bioresour. Technol..

[41] Daraghmeh O.A., Jensen J.R., Petersen C.T. (2009). Soil Structure Stability under Conventional and Reduced Tillage in a Sandy Loam. Geoderma.

[42] Huang R., Lan M., Liu J., Gao M. (2017). Soil Aggregate and Organic Carbon Distribution at Dry Land Soil and Paddy Soil: The Role of Different Straws Returning. Environ. Sci. Pollut. Res..

[43] Zhang X., Xin X., Yang W., Zhu A., Ding S. (2019). Short-Term Decomposition, Turnover and Retention of Residue-Derived Carbon Are Influenced by the Fertility Level in a Sandy Loam Soil. Geoderma.

[44] Garcia L., Damour G., Gary C., Follain S., Le Bissonnais Y., Metay A. (2019). Trait-Based Approach for Agroecology: Contribution of Service Crop Root Traits to Explain Soil Aggregate Stability in Vineyards. Plant Soil.

[45] Le Bissonnais Y., Prieto I., Roumet C., Nespoulous J., Metayer J., Huon S., Villatoro M., Stokes A. (2018). Soil Aggregate Stability in Mediterranean and Tropical Agro-Ecosystems: Effect of Plant Roots and Soil Characteristics. Plant Soil.

[46] Zheng F., Liu X., Ding W., Song X., Li S., Wu X. (2023). Positive Effects of Crop Rotation on Soil Aggregation and Associated Organic Carbon Are Mainly Controlled by Climate and Initial Soil Carbon Content: A Meta-Analysis. Agric. Ecosyst. Environ..

[47] Niu Z., An F., Su Y., Li J., Liu T. (2024). Effects of Cropping Patterns on the Distribution, Carbon Contents, and Nitrogen Contents of Aeolian Sand Soil Aggregates in Northwest China. Sci. Rep..

[48] Bu R., Ren T., Lei M., Liu B., Li X., Cong R., Zhang Y., Lu J. (2020). Tillage and Straw-Returning Practices Effect on Soil Dissolved Organic Matter, Aggregate Fraction and Bacteria Community under Rice-Rice-Rapeseed Rotation System. Agric. Ecosyst. Environ..

[49] Cheng Z., Guo J., Jin W., Liu Z., Wang Q., Zha L., Zhou Z., Meng Y. (2024). Responses of SOC, Labile SOC Fractions, and Amino Sugars to Different Organic Amendments in a Coastal Saline-Alkali Soil. Soil Tillage Res..

[50] Tian X., Wang C., Bao X., Wang P., Li X., Yang S., Ding G., Christie P., Li L. (2019). Crop Diversity Facilitates Soil Aggregation in Relation to Soil Microbial Community Composition Driven by Intercropping. Plant Soil.

[51] Kihara J., Martius C., Bationo A., Thuita M., Lesueur D., Herrmann L., Amelung W., Vlek P.L.G. (2012). Soil Aggregation and Total Diversity of Bacteria and Fungi in Various Tillage Systems of Sub-Humid and Semi-Arid Kenya. Appl. Soil Ecol..

[52] Duan Y., Chen L., Li Y., Wang Q., Zhang C., Ma D., Li J., Zhang J. (2021). N, P and Straw Return Influence the Accrual of Organic Carbon Fractions and Microbial Traits in a Mollisol. Geoderma.

[53] Six J., Bossuyt H., Degryze S., Denef K. (2004). A History of Research on the Link between (Micro)Aggregates, Soil Biota, and Soil Organic Matter Dynamics. Soil Tillage Res..

[54] Wu H., Cui H., Fu C., Li R., Qi F., Liu Z., Yang G., Xiao K., Qiao M. (2024). Unveiling the Crucial Role of Soil Microorganisms in Carbon Cycling: A Review. Sci. Total Environ..

[55] Srivastava P., Singh R., Bhadouria R., Tripathi S., Raghubanshi A.S. (2020). Temporal Change in Soil Physicochemical, Microbial, Aggregate and Available C Characteristic in Dry Tropical Ecosystem. CATENA.

[56] Schlatter D.C., DavelosBaines A.L., Xiao K., Kinkel L.L. (2013). Resource Use of Soilborne *Streptomyces* Varies with Location, Phylogeny, and Nitrogen Amendment. Microb. Ecol..

[57] Yang K., Peng P., Duan F., Tang H., Wu K., Wu Z., Li F., Chen Y., Zou C., Liu L. (2023). Microbial Mechanisms of the Priming Effect over 12 Years of Different Amounts of Nitrogen Management. Agronomy.

[58] Zheng X., Dai X., Zhu Y., Yang J., Jiang H., Dong H., Huang L. (2022). (Meta)Genomic Analysis Reveals Diverse Energy Conservation Strategies Employed by Globally Distributed Gemmatimonadota. mSystems.

[59] Chen W.-C., Ko C.-H., Su Y.-S., Lai W.-A., Shen F.-T. (2021). Metabolic Potential and Community Structure of Bacteria in an Organic Tea Plantation. Appl. Soil Ecol..

[60] Yuan Q., Wang P., Wang X., Hu B., Tao L. (2022). Phytoremediation of Cadmium-Contaminated Sediment Using *Hydrilla verticillata* and *Elodea canadensis* Harbor Two Same Keystone Rhizobacteria *Pedosphaeraceae* and *Parasegetibacter*. Chemosphere.

[61] Insuk C., Pongpamorn P., Forsythe A., Matsumoto A., Ōmura S., Pathom-aree W., Cheeptham N., Xu J. (2022). Taxonomic and Metabolite Diversities of Moss-Associated Actinobacteria from Thailand. Metabolites.

[62] Pokluda R., Ragasova L., Jurica M., Kalisz A., Komorowska M., Niemiec M., Sekara A. (2021). Effects of Growth Promoting Microorganisms on Tomato Seedlings Growing in Different Media Conditions. PLoS ONE.

[63] Degelmann D.M., Kolb S., Dumont M., Murrell J.C., Drake H.L. (2009). Enterobacteriaceae Facilitate the Anaerobic Degradation of Glucose by a Forest Soil. FEMS Microbiol. Ecol..

[64] de Andrade da Silva M.S.R., de Melo Silveira dos Santos B., de Andrade da Silva C.S.R., de Andrade da Silva C.S.R., de Sousa Antunes L.F., dos Santos R.M., Santos C.H.B., Rigobelo E.C. (2021). Humic Substances in Combination With Plant Growth-Promoting Bacteria as an Alternative for Sustainable Agriculture. Front. Microbiol..

[65] Bondareva L., Kudryasheva N. (2021). Direct and Indirect Detoxification Effects of Humic Substances. Agronomy.

[66] Wang Y., Fan Y., Wang Q., Zhang S., Shi Y., Zheng X. (2022). Response of Soil Fertility and Bacterial Community Composition to Vegetation Species in a Coal Mining Subsidence Area: A Survey After 20-Year Reclamation. Front. Environ. Sci..

[67] Wang Y., Zhong W., Zhang X., Cao M., Ni Z., Zhang M., Li J., Duan Y., Wu L. (2024). Copper Pyrazole Addition Regulates Soil Mineral Nitrogen Turnover by Mediating Microbial Traits. Front. Microbiol..

[68] Cao M., Duan Y., Li M., Cai-guo T., Kan W., Li J., Zhang H., Zhong W., Wu L. (2024). Manure Substitution Improves Maize Yield by Promoting Soil Fertility and Mediating the Microbial Community in Lime Concretion Black Soil. J. Integr. Agric..

[69] Zhang D., Xing Y., Wang X., Li W., Guo Y., Tang Y., Zhang H., Chen J., Jiang B. (2024). The Effect of Polyvinyl Chloride Microplastics on Soil Properties, Greenhouse Gas Emission, and Element Cycling-Related Genes: Roles of Soil Bacterial Communities and Correlation Analysis. J. Hazard. Mater..

[70] Thomas L., Ram H., Kumar A., Singh V.P. (2016). Production, Optimization, and Characterization of Organic Solvent Tolerant Cellulases from a Lignocellulosic Waste-Degrading Actinobacterium, *Promicromonospora* Sp. VP111. Appl. Biochem. Biotechnol..

[71] Thomas L., Ram H., Singh V.P. (2023). Multipurpose Cellulases of *Promicromonospora* Sp. VP111, with Broad Substrate Specificity and Tolerance Properties. J. Basic Microbiol..

[72] Lee R.M., Griffin N., Jones E., Abbott B.W., Frei R.J., Bratsman S., Proteau M., Errigo I.M., Shogren A., Bowden W.B. (2022). Bacterioplankton Dispersal and Biogeochemical Function across Alaskan Arctic Catchments. Environ. Microbiol..

