# Supplementary material for: Long-Term Crop Rotation Revealed the Relationship Between Soil Organic Carbon Physical Fraction and Bacterial Community at Aggregate Scales
Source: Microorganisms. 2025 Feb 24;13(3):496. doi: 10.3390/microorganisms13030496 (PMC11944754; doi:10.3390/microorganisms13030496)
Supplement: Supplementary file 1 [file microorganisms-13-00496-s001.zip › microorganisms-3450629-supplementary.pdf]

Title: Long-Term Crop Rotation Revealed the Relationship Between Soil Organic Carbon Physical Fraction and Bacterial Community at Aggregate Scales

Authors name: Xianghai Meng [1], Baicheng Wang [1], Xingzhe Zhang [1], Chunguang Liu [1], Jinghong Ji [2], Xiaoyu Hao [2], Bing Yang [1], Wenhui Wang [1], Dehai Xu [1], Shuai Zhang [1], Xiaomei Wang [1], Minghui Cao [3,*] and Yuming Wang [3,4,*]

1 Mudanjiang Branch, Heilongjiang Academy of Agricultural Sciences, Mudanjiang 157000, China; mengxianghai538@163.com (X.M.); 13946368993@163.com (B.W.); jxnczxz@163.com (X.Z.); 18045341845@163.com (C.L.); yangb19961112@163.com (B.Y.); wangwenhui1411@126.com (W.W.); 13946350000@163.com (D.X.); 14311@163.com (S.Z.); wxmaiwyp2006@126.com (X.W.)

2 Heilongjiang Academy of Black Soil Conservation & Utilization, Harbin 150086, China; jinghong_98@163.com (J.J.); xiaoyuhao1981@sina.com (X.H.)

3 The Centre for Ion Beam Bioengineering Green Agriculture, Hefei Institutes of Physical Science, Chinese Academy of Sciences, Hefei 230031, China

4 Science Island Branch, Graduate School, University of Science and Technology of China, Hefei 230026, China

* Correspondence: mhcao@iim.ac.cn (M.C.); wym199702@163.com (Y.W.)

Supplementary material

Text S1 Soil aggregate isolation and SOC fraction determination

The soil samples were physically separated into three aggregate fractions according to the methods described by Six et al. [31]. All the samples were broken apart gently into small pieces along natural break points. The soil samples were placed on top of a 0.25 mm sieve and submerged in deionized water for approximately 5 min. Then, they were separated at an amplitude of 3 cm and a frequency of 30 cycles per min for a duration of 2 min. After shaking, the aggregate subsamples above each sieve were collected as follows: macroaggregates (0.25–2 mm), microaggregates (0.053–0.25 mm), and clay and silt fractions (0–0.053 mm). After wet sieving, all the aggregates were immediately centrifuged at 2000 r min^{-1} for 2 min, freeze-dried and weighed.

The soil POC was determined using the method described by Yu et al. [32]. Macroaggregates and microaggregates were further fractionated into POC and MaOC. In general, 5.0 g (dry weight) of aggregate was mixed with 0.5% sodium hexametaphosphate solution, and the samples were centrifuged at 200 rpm for 18 h. The samples were passed through 250-μm (POC) and 53-μm sieves (MaOC). All aggregate C levels were measured using $K_2Cr_2O_7$ digestion [30].

Text S2 High-throughput sequencing

High-throughput sequencing was performed with the Illumina MiSeq sequencing platform (Illumina, Inc.). The primers, 515F (5′- GTGCCAGCMGCCGCGGTAA-3′) and 907R (5′-CCGTCAATTCMTTTRAGTTT-3′), were chosen to amplify the 16S rRNA genes in the V4–V5 hypervariable region. A unique 5-bp barcode sequence was added to the forward primers to distinguish the PCR products from different samples. PCR was conducted in a 50-μL reaction mixture containing 27 μL of ddH2O, 2 μL (5 μM) of each forward/reverse primer, 2.5 μL (10 ng) of template DNA, 5 μL (2.5 mM) of deoxynucleoside triphosphates, 10 μL of 5 × FastPfu buffer, 0.5 μL of bovine serum albumin, and 1 μL of TransStart FastPfu polymerase (TransGen, Beijing, China). The PCR conditions were 94°C for 5 min; 30 cycles of 94°C for 30 s, 52°C for 30 s and 72°C for 30 s of extension; and 72°C for 10 min. The reaction products were pooled and purified using a QIAquick PCR Purification Kit (Qiagen), and they were quantified via a NanoDrop ND-1000 spectrophotometer (Thermo Scientific). After the individual quantification step, amplicons were pooled in equal amounts, and paired-end 2300-bp sequencing was performed via the Illumina MiSeq platform with the MiSeq Reagent Kit v3. The PCR products from all the samples were pooled and purified at equimolar concentrations, and sequencing was performed on an Illumina MiSeq instrument [33].

Table S1 The soil basic property under different treatments

Treatments	Soil pH	SOC (g/kg)	TN (g/kg)	TP (g/kg)	C: N	C: P	N: P
CK	7.01±0.02 b	12.65±0.90 b	1.72±0.12 a	1.23±0.02 a	7.35±0.05 b	10.31±0.61 b	1.40±0.08 b
MM	7.44±0.02 a	15.21±0.70 ab	1.53±0.13 a	0.76±0.01 c	10.01±1.26 ab	19.92±0.80 a	2.01±0.18 a
SS	7.06±0.02 b	13.53±0.97 b	1.67±0.13 a	1.16±0.01 a	8.12±0.37 b	11.66±0.82 b	1.44±0.11 b
MS	7.48±0.03 a	16.74±1.09 a	1.53±0.14 a	0.84±0.01 b	11.02±1.23 a	20.02±1.44 a	1.83±0.17 b

The results show means ± standard deviations (n = 3). Different lowercase letters after values indicate significant differences between each treatment, $P < 0.05$. CK, pre-treatment; MM, maize monoculture; SS, soybean monoculture; MS, maize-soybean rotation.

Table S2 The soil microbial shannon index under different treatments

Groups	Macroaggregate	Microaggregate
MM	9.62±0.41a	9.09±1.02a
SS	9.62±0.35a	9.35±0.72a
MS	9.62±0.07a	8.68±0.23a

The results show means ± standard deviations (n = 3). Different lowercase letters after values indicate significant differences between each treatment, $P < 0.05$. MM, maize monoculture; SS, soybean monoculture; MS, maize-soybean rotation.

Table S3 The information of selected bacterial taxa within macroaggregate

Id	Cluster	Phylum	Family	Genus
ASV246	1	Actinobacteriota	Solirubrobacteraceae	Solirubrobacter
ASV332	1	Chloroflexi	S085	S085
ASV252	1	Actinobacteriota	Streptomycetaceae	Streptomyces
ASV28	1	Gemmatimonadota	Gemmatimonadaceae	
ASV76	4	Proteobacteria	Xanthomonadaceae	
ASV159	1	Actinobacteriota	Solirubrobacteraceae	Solirubrobacteraceae
ASV131	3	Verrucomicrobiota	Pedosphaeraceae	
ASV233	1	Acidobacteriota	Vicinamibacteraceae	Vicinamibacteraceae
ASV418	3	Gemmatimonadota	Gemmatimonadaceae	
ASV460	3	Acidobacteriota		

Table S4 The information of selected bacterial taxa within microaggregate

Id	Cluster	Phylum	Family	Genus
ASV144	2	Actinobacteriota	Pseudonocardiaceae	Saccharothrix
ASV127	2	Actinobacteriota	MB-A2-108	MB-A2-108
ASV328	2	Actinobacteriota	Solirubrobacteraceae	
ASV382	2	Actinobacteriota	Mycobacteriaceae	Mycobacterium
ASV186	1	Actinobacteriota	Ilumatobacteraceae	
ASV296	1	Proteobacteria	Rhizobiales_Incertae_Sedis	Nordella
ASV370	2	Actinobacteriota		
ASV838	2	Proteobacteria	Pseudomonadaceae	Pseudomonas
ASV81	1	Chloroflexi	Gitt-GS-136	Gitt-GS-136
ASV176	1	Chloroflexi	JG30-KF-CM45	JG30-KF-CM45
ASV300	1	Proteobacteria	Methylophilaceae	Methylotenera
ASV396	1	Actinobacteriota	Microbacteriaceae	
ASV695	2	Proteobacteria	Enterobacteriaceae	
ASV810	1	Actinobacteriota	Nocardioidaceae	
ASV367	2	Actinobacteriota	67-14	67-14
ASV124	2	Actinobacteriota	Pseudonocardiaceae	Actinophytocola
ASV138	4	Actinobacteriota		
ASV247	1	Actinobacteriota	Rubrobacteriaceae	Rubrobacter
ASV293	1	Proteobacteria	Devosiaceae	Devosia
ASV527	2	Actinobacteriota	Geodermatophilaceae	Blastococcus
ASV400	2	Proteobacteria	Caulobacteraceae	PMMR1
ASV405	4	Proteobacteria	Nitrosomonadaceae	Ellin6067

Table S5 The information of microbial keystone taxa

ID	Cluster	Phylum	Class	Order	Family	Genus	Species
ASV231	4	Gemmatimonadota	Gemmatimonadetes	Gemmatimonadales	Gemmatimonadaceae		
ASV317	1	Acidobacteriota	Vicinamibacteria	Vicinamibacterales	Vicinamibacteraceae	Vicinamibacteraceae	
ASV66	4	Acidobacteriota	Acidobacteriae	Bryobacterales	Bryobacteraceae	Bryobacter	
ASV108	2	Chloroflexi	KD4-96	KD4-96	KD4-96	KD4-96	
ASV109	2	Acidobacteriota	Blastocatellia	Pyrinomonadales	Pyrinomonadaceae	RB41	
ASV303	2	Actinobacteriota	Acidimicrobiia				
ASV305	2	Actinobacteriota	Thermoleophilia	Gaiellales	Gaiellaceae	Gaiella	Actinobacterium

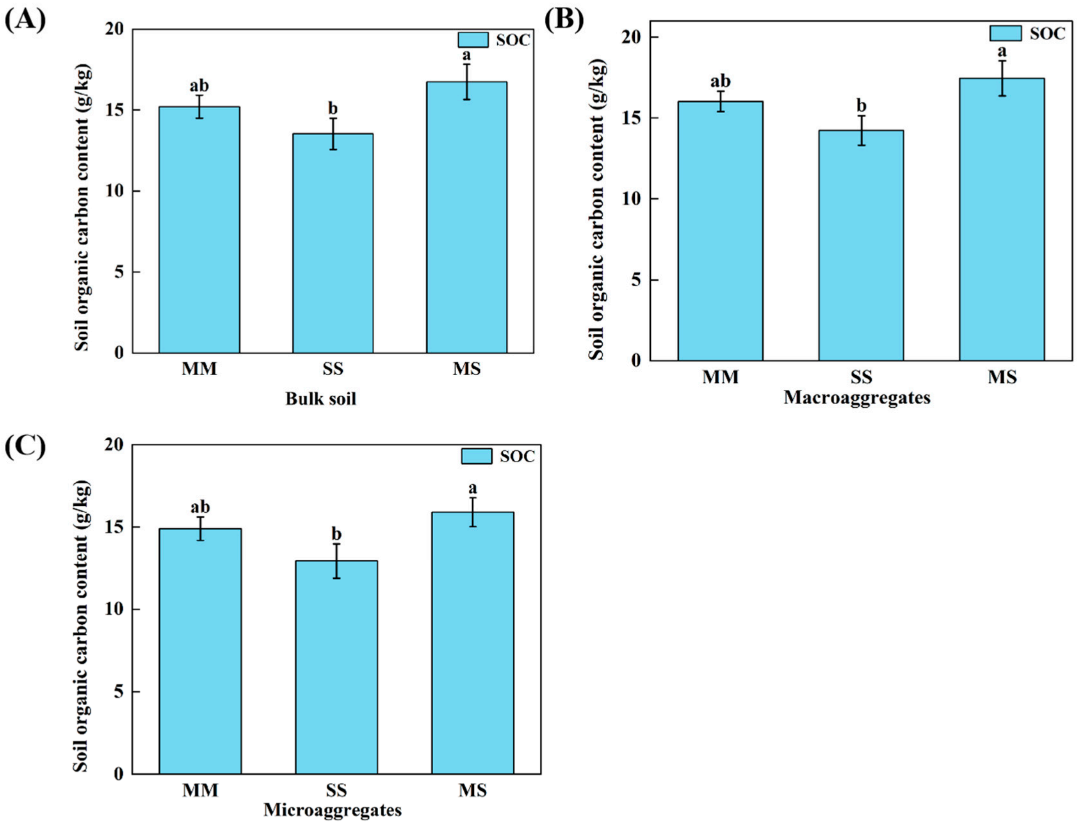

Figure S1 Soil organic carbon content in bulk soil (A), macroaggregate (B) and microaggregate (C). SOC, soil organic carbon. MM, maize monoculture; SS, soybean monoculture; MS, maize-soybean rotation

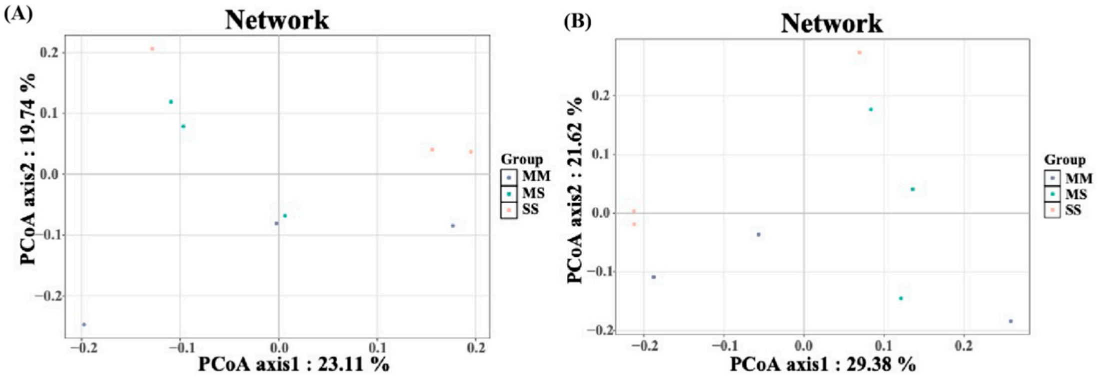

Figure S2 PCoA showing the bacteria within macroaggregate (A) and microaggregate (B) under different treatments. MM, maize monoculture; SS, soybean monoculture; MS, maize-soybean rotation.

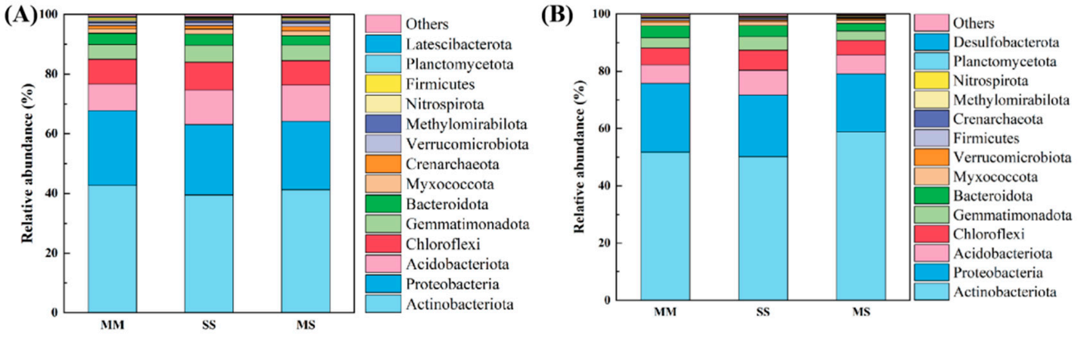

Figure S3 Soil microbial community compositions within macroaggregate (A) and microaggregate (B) under different treatments. MM, maize monoculture; SS, soybean monoculture; MS, maize-soybean rotation.
